# The Acceptability of Text Messaging to Help African American Women Manage Anxiety and Depression: Cross-Sectional Survey Study

**DOI:** 10.2196/15801

**Published:** 2020-02-03

**Authors:** Terika McCall, Todd A Schwartz, Saif Khairat

**Affiliations:** 1 Carolina Health Informatics Program University of North Carolina at Chapel Hill Chapel Hill, NC United States; 2 School of Nursing University of North Carolina at Chapel Hill Chapel Hill, NC United States; 3 Department of Biostatistics Gillings School of Global Public Health University of North Carolina at Chapel Hill Chapel Hill, NC United States

**Keywords:** African Americans, women, anxiety, depression, mHealth, text messaging

## Abstract

**Background:**

The rates of mental illness among African American women are comparable with the general population; however, they significantly underutilize mental health services compared with their white counterparts. Previous studies revealed that interventions delivered via text messaging are effective and can be used to increase access to services and resources. More insight into whether or not this modality is acceptable for use to deliver mental health care to help African American women manage anxiety and depression is needed.

**Objective:**

This exploratory study aimed to gain insight into the acceptability of using text messaging to help African American women manage anxiety and depression.

**Methods:**

A self-administered Web-based survey was launched in June 2018 and closed in August 2018. Eligible participants were African American women (18 years or older) who reside in the United States. Participants were recruited through convenience sampling (eg, email sent via listservs and social media posts). Respondents were provided an anonymous link to the questionnaire. The survey consisted of 53 questions on the following subjects: sociodemographic characteristics, attitudes toward seeking professional psychological help, mobile phone use, and acceptability of using a mobile phone to receive mental health care.

**Results:**

The results of this exploratory study (N=101) showed that fewer than half of respondents endorsed the use of text messaging to communicate with a professional to receive help to manage anxiety (49/101, 48.5%) and depression (43/101, 42.6%). Approximately 51.4% (52/101) agreed that having the option to use text messaging to communicate with a professional if they are dealing with anxiety would be helpful. Similarly, 48.5% (49/101) agreed that having the option to use text messaging to communicate with a professional if they are dealing with depression would be helpful. Among participants who agreed that text messaging would be helpful, more than 80% noted being comfortable with its use to receive help for managing anxiety (approximately 86%, 45/52) and depression (approximately 82%, 40/49; highly significant positive association, all *P*<.001). More than 50% of respondents (56/101, 55.4%) indicated having concerns about using text messaging. No statistically significant associations were found between age and agreement with the use of text messaging to communicate with a professional to receive help for managing anxiety (*P*=.26) or depression (*P*=.27).

**Conclusions:**

The use of text messaging was not highly endorsed by African American women as an acceptable mode of communication with a professional to help them manage anxiety or depression. Concerns around privacy, confidentiality, and the impersonal feel of communicating about sensitive issues via text messages must be addressed for this modality to be a viable option. The findings of this study demonstrated the need for further research into the use of mobile technology to provide this population with more accessible and convenient options for mental health care.

## Introduction

African American women experience rates of mental illness comparable to the general population (18.6% vs 18.9%) [1]. However, they use mental health services at less than half the rate of their white counterparts (10.6% compared with 23.4%) [1]. Approximately 16% of non-Hispanic black women reported having generalized anxiety in their lifetime [2]. Furthermore, 27% of non-Hispanic black women reported experiencing depression in their lifetime [2]. Historically, mental illness has been underreported in the African American community; therefore, the true burden may actually be significantly higher than reported prevalence estimates.

More than 64% of African American women who reported experiencing mental illness in the last year did not receive any mental health treatment during that time [1]. A study by Watson and Hunter [3] explored the attitudes and perceptions of African American women toward professional help seeking for mental health services and found that they “held less favorable attitudes toward professional help-seeking than previous, non-African American samples.” There are many reasons why African American women may not seek mental health services when needed. Barriers such as stigmatization of mental illness, less access to treatment, no or inadequate health insurance, mistrust of providers, and low health literacy prevent traditionally marginalized populations from seeking care [4,5].

Evidence from previous studies showed that telehealth interventions for anxiety [6-13] and depression [7,9-20] are effective. Previous interventions have used modalities such as telephone [9,16], videoconferencing [11,17], text messaging [18,20], Web-based formats (eg, websites and email) [6,15,21], and mobile-optimized websites and apps [13,19] to help participants reduce anxiety or depressive symptoms. The convenience and familiarity of using telehealth modalities (eg, text messaging), coupled with the use of proven psychotherapy treatments, such as cognitive behavioral therapy (CBT), make telemental health interventions suitable alternatives to traditional in-person treatment. Furthermore, mobile health (mHealth) interventions have been increasingly used because of the potential to reduce access issues, such as geographic proximity to a preferred mental health care professional (eg, therapist). Previous studies have shown that African American women are comfortable with participating in mHealth research and interventions [22,23], and 80% of African American women own smartphones [24]. This presents a great opportunity to use mobile technology to help reduce the disparity in mental health service utilization and improve health outcomes for this population.

Previous literature reviews have found that, overall, text messaging is effective in improving mental health–related outcomes, treatment adherence, and appointment attendance [25,26]. However, the majority of the published studies were conducted with predominantly white study samples. Therefore, the results may not be generalizable to all racial groups. To our knowledge, there have been no studies that have examined the acceptability of text messaging to help African American women manage anxiety or depression. Nonetheless, prior studies that included a representative sample of African American participants (>13% of the study sample) and used text messaging for weight management, physical activity, or prenatal care education interventions have been effective [27-30]. Therefore, the insufficient representation of African Americans in previous telemental health studies may be largely because of ineffective recruitment and retention strategies, in addition to the previously discussed barriers that prevent them from seeking mental health care [31,32].

Owing to the scarcity of studies that include a significant representation of African American women in the sample and the underutilization of mental health services by this population, the population should be surveyed to determine the acceptability of using text messaging for mental health care. The aim of this exploratory study was to gain insight into the acceptability of using text messaging to help African American women manage anxiety and depression, specifically comfortability with using text messaging to communicate with a professional to receive help to manage anxiety and depression.

## Methods

### Study Design and Recruitment

The Web-based questionnaire was opened in June 2018 and closed in August 2018. Women (18 years or older) who identify as African American and reside within the United States, regardless of mental health history, were eligible to participate. Participants were recruited through convenience sampling. Recruitment methods included receiving an invitation to take the survey via a direct email from the first author or email sent through listservs whose membership is primarily African American women or solicitation via social media posts (eg, posts in Facebook groups) or direct messages. A research information sheet about the study was provided via a link in the email text or social media posts. Following the snowball sampling method, respondents were encouraged to share the link to the survey with their networks (eg, family, friends, and professional organizations). No remuneration was offered for participation. The Institutional Review Board of the University of North Carolina at Chapel Hill provided the study a notification of exemption from further review.

### Measures

The computer-assisted Web interviewing data collection method was used to administer the survey because of the sensitive nature of the questions and to reduce respondent burden. Respondents were provided an anonymous link to the Web-based questionnaire. No personally identifiable information (PII) was collected in the survey. The survey was self-administered using Qualtrics software. The 53 questions included in the survey covered the following domains: sociodemographic characteristics, attitudes toward seeking professional psychological help, mobile phone use, and acceptability of using a mobile phone to receive mental health care.

Questions about sociodemographic characteristics, such as the respondent’s race, ethnicity, age, gender, and highest level of education attained, were asked at the beginning of the survey. The race, age, and gender questions were used as screener questions to determine eligibility to continue the survey. If the respondent did not self-identify as African American (or biracial, African American, and another race), female, and 18 years or older, they were routed directly to the end of the survey.

#### Attitudes Toward Seeking Professional Psychological Help

Respondents’ attitudes toward seeking professional psychological help were measured using questions from an adapted version of the validated *Inventory of* *Attitudes Toward Seeking Mental Health Services* (IASMHS) [33]. The IASMHS consists of 24 questions that contribute to a total IASMHS score and the following factors: psychological openness, help-seeking propensity, and indifference to stigma. Response options to the survey items were on a 5-point Likert-type scale ranging from 0 (disagree) to 4 (agree). Before data analysis, all negatively worded items were reverse coded.

In the survey, the term *professional* referred to individuals who have been trained to deal with mental health problems (eg, psychologists, psychiatrists, social workers, and family physicians). To collect data specifically about attitudes toward seeking professional help for managing anxiety and depression, 6 questions in the inventory were revised. In these 6 questions, the words *psychological* *problems* or *mental disorder* were substituted with *anxiety* and then repeated for substitution with *depression*. For example, item #16 in the IASMHS reads, “I would be uncomfortable seeking professional help for psychological problems because people in my social or business circles might find out about it.” The 2 corresponding revised survey questions state, “I would be uncomfortable seeking professional help for *anxiety* because people in my social or business circles might find out about it” and “I would be uncomfortable seeking professional help for *depression* because people in my social or business circles might find out about it.” This increased the total number of questions in the inventory to 30 and permitted calculation of a total IASMHS score related to anxiety; a total IASMHS score related to depression; and subscores for psychological openness, help-seeking propensity, indifference to stigma for anxiety, and indifference to stigma for depression. Scores on the IASMHS range from 0 to 96, with subscale scores ranging from 0 to 32. Higher scores indicate more positive attitudes toward seeking professional psychological help.

#### Acceptability of the Use of Text Messaging for Mental Health Care

The use of text messaging was ascertained with the following items: (1) current mobile phone ownership (yes/no) and (2) frequency of sending text messages (never, <1 time per week, 1-6 times per week, 1-3 times per day, and ≥4 times per day). Acceptability of using text messaging to receive help to manage anxiety or depression was measured by response to the statements, “I would feel comfortable communicating with a professional through text messaging to receive help for managing *anxiety*” and “I would feel comfortable communicating with a professional through text messaging to receive help for managing *depression*.” Respondents were also asked about the perceived helpfulness of having the option to use text messaging to communicate with a professional if they are feeling anxious or depressed. Perceived helpfulness was gauged by response to the statements, “Having the option to use text messaging to communicate with a professional if I am dealing with *anxiety* would be helpful for me” and “Having the option to use text messaging to communicate with a professional if I am dealing with *depression* would be helpful for me.” Response options to the survey items were on a 5-point Likert-type scale ranging from 0 (disagree) to 4 (agree). Before completing the survey, respondents were asked, “Do you have any concerns about using text messaging to communicate with a professional?” If they answered “Yes” to this question, they were presented with an open-ended question asking them to note their concerns in the textbox provided.

### Statistical Analysis

#### Quantitative Data Analysis

Descriptive statistics were calculated for sample characteristics and responses to text messaging questions as mean, standard deviation, and range for continuous variables and as frequencies and percentages for categorical variables. As reported in prior work, age was dichotomized into 2 groups (<50 years and ≥50 years), education was categorized into 3 levels (less than bachelor’s degree, bachelor’s degree, and graduate degree), and response options were dichotomized as agree/somewhat agree and disagree/somewhat disagree [22]. Fisher exact test was used to determine whether an association exists between the response to each text messaging question and age group and to test for association between agreement with comfortability and perceived helpfulness of having the option to communicate with a professional through text messaging to receive help for managing anxiety and depression, respectively. Independent groups *t* tests were separately performed to assess group differences in mean scores for each on psychological openness, help-seeking propensity, indifference to anxiety stigma, indifference to depression stigma, and IASMHS scores for anxiety and depression, respectively, between the participants who agreed with the use of text messaging to communicate with a professional to receive help to manage anxiety and depression and those who disagreed.

Furthermore, a sensitivity analysis was performed using independent groups *t* tests to assess group differences in mean scores, in the aforementioned categories, between the participants who agreed (agree/somewhat agree) with the use of text messaging to communicate with a professional to receive help to manage anxiety and depression and those who did not indicate agreement (disagree/somewhat disagree/undecided). *Undecided* responses were included to see whether the statistical significance changed. Statistical significance was determined at the 2-sided *P*<.05 level for all tests. Statistical analyses were conducted using SPSS version 25 software.

#### Qualitative Data Analysis

Thematic analysis was conducted on responses to the question, “What are your concerns about using text messaging to communicate with a professional?” The responses were imported into NVivo 12 for analysis. The data were categorized by TM and SK reading through each response and coding the emerging themes. Responses could be assigned as many themes as were pertinent.

## Results

### Participants

The characteristics of the study participants are summarized in Table 1. Out of the 113 respondents who started the survey, 102 completed it (90.3% completion rate). Of the 102 respondents, 1 was removed because of item nonresponse, providing an analysis sample of 101 participants. Participants ranged in age from 19 to 80 years (mean age 38.9 [SD 13.2] years), and all participants identified as African American or biracial (ie, African American and another race) and female. Most respondents (99/101, 98.0%) identified as non-Hispanic. Approximately 15% (15/101) of respondents had less than a bachelor’s degree, 23.8% (24/101) obtained a bachelor’s degree, and 61.4% (62/101) had a graduate degree. All participants reported the use of text messaging, and 90.1% (91/101) of participants indicated texting 4 or more times per day.

**Table 1:** Characteristics of study participants (N=101).

Characteristics		Values
Age (years), mean (SD)		38.9 (13.2)
**Age group (years), n (%)**		
	<50	80 (79.2)
	≥50	21 (20.8)
**Race, n (%)**		
	African American	99 (98.0)
	Biracial^a^	2 (2.0)
**Ethnicity, n (%)**		
	Hispanic	2 (2.0)
	Non-Hispanic	99 (98.0)
**Education^b^, n (%)**		
	Less than bachelor’s degree	15 (14.9)
	Bachelor’s degree	24 (23.8)
	Graduate degree	62 (61.4)
**Frequency of using text messaging, n (%)**		
	1-6 times per week	2 (2.0)
	1-3 times per day	8 (7.9)
	≥4 times per day	91 (90.1)

^a^Biracial defined as identifying as African American and another race.

^b^Percentages may not sum to 100% because of rounding.

### Communicating With a Professional Via Text Messaging

The results of this exploratory study showed that less than half of respondents endorsed the use of text messaging to communicate with a professional to receive help to manage anxiety and depression. Only 48.5% (49/101) of respondents indicated agreement (26/101, 25.7% agree and 23/101, 22.8% somewhat agree), 10.9% (11/101) were undecided, and 40.6% (41/101) showed disagreement (26/101, 25.7% disagree and 15/101, 14.9% somewhat disagree) with the statement, “I would feel comfortable communicating with a professional through text messaging to receive help for managing *anxiety*.” Similarly, 42.6% (43/101) of respondents indicated agreement (23/101, 22.8% agree and 20/101, 19.8% somewhat agree), 14.9% (15/101) were undecided, and 42.5% (43/101) showed disagreement (26/101, 25.7% disagree and 17/101, 16.8% somewhat disagree) with the statement, “I would feel comfortable communicating with a professional through text messaging to receive help for managing *depression*.”

**Figure 1:**
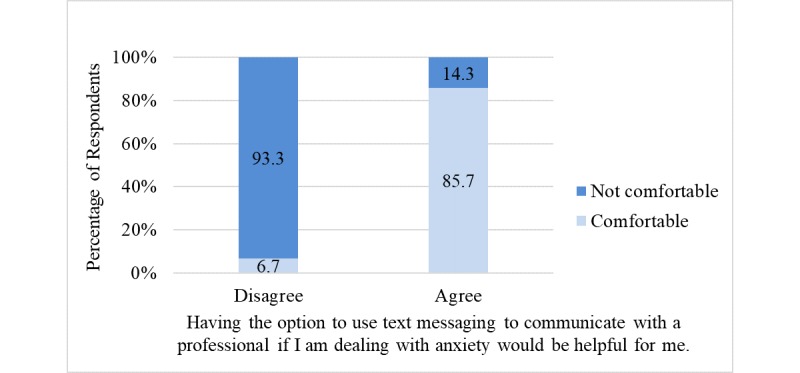
Sample percentages showing the bivariate relationship between perceived helpfulness and comfortability with using text messaging to communicate with a professional to receive help to manage anxiety (*P*&lt;.001).

**Figure 2:**
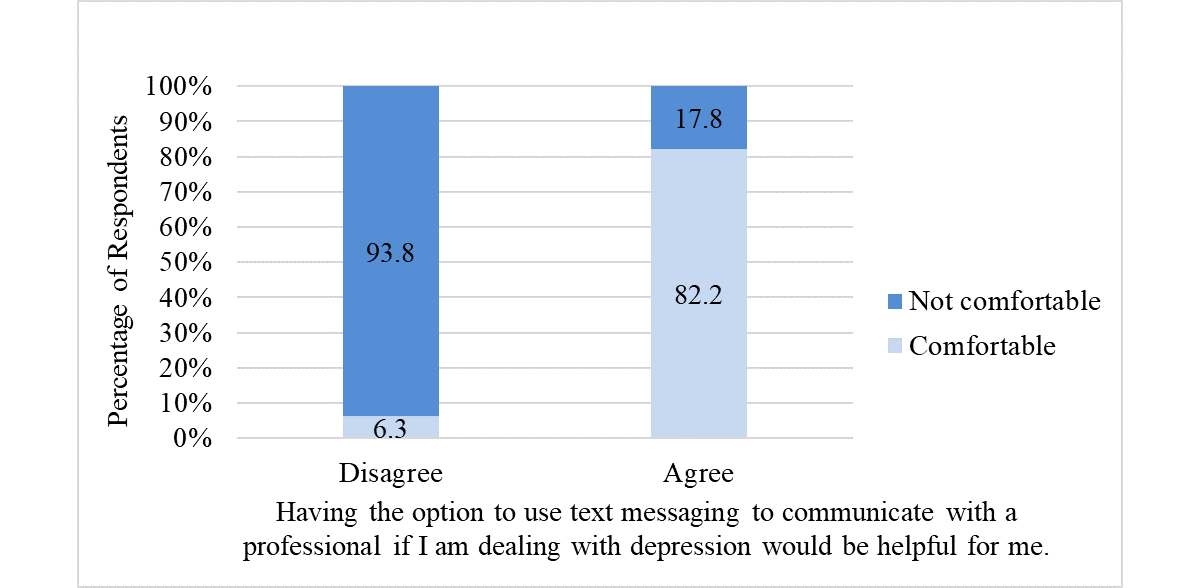
Sample percentages showing the bivariate relationship between perceived helpfulness and comfortability with using text messaging to communicate with a professional to receive help to manage depression (*P*&lt;.001). Note: percentages may not sum to 100% because of rounding.

Approximately 51% (52/101) of respondents agreed that having the option to use text messaging to communicate with a professional if they are dealing with anxiety would be helpful. Similarly, 48.5% (49/101) of respondents agreed that having the option to use text messaging to communicate with a professional if they are dealing with depression would be helpful. Figures 1 and 2 illustrate the bivariate relationship between perceived helpfulness and comfortability with having the option to communicate with a professional via text messaging to receive help dealing with anxiety and depression, respectively.

Among participants who agreed that text messaging would be helpful, approximately 86% (45/52) noted being comfortable with its use to receive help for managing anxiety (highly significant positive association; *P*<.001); in contrast, among participants who disagreed that text messaging would be helpful, approximately 7% (3/49) noted being comfortable with its use to receive help for managing anxiety (Figure 1). Of those who agreed with the statement, “Having the option to use text messaging to communicate with a professional if I am dealing with *depression* would be helpful for me,” approximately 82% (40/49) indicated being comfortable with its use to receive help for managing depression (highly significant positive association; *P*<.001); however, among participants who disagreed that text messaging would be helpful, approximately 6% (3/52) indicated being comfortable with its use to receive help for managing depression (Figure 2). No statistically significant associations were found between age and agreement with the use of text messaging to communicate with a professional to receive help for managing anxiety (*P*=.26) or depression (*P*=.27). Furthermore, no statistically significant association was found between age and response to the question, “Do you have any concerns about using text messaging to communicate with a professional?” (*P*>.99).

### Attitudes Toward Seeking Mental Health Service and Acceptance of Text Messaging

The study participants held favorable views toward seeking mental health services. Respondents’ reports of psychological openness (mean 23.95, SD 4.53) and help-seeking propensity (mean 26.11, SD 4.89) were comparable with the adult female normative scores for psychological openness (mean 23.19, SD 6.00) and help-seeking propensity (mean 24.95, SD 4.74) [33]. The indifference to stigma questions were adapted to collect data on indifference to sigma for anxiety and depression. The participants’ scores on indifference to anxiety stigma (mean 24.34, SD 6.08) and depression stigma (mean 23.58, SD 6.43) were similar.

Tables 2 and 3 display group IASMHS factor scores by the level of agreement with using text messaging to communicate with a professional to receive help for managing anxiety and depression, respectively. There were no statistically significant differences between group mean scores for psychological openness (*P*=.96), help-seeking propensity (*P*=.68), indifference to anxiety stigma (*P*=.28), and IASMHS scores (*P*=.47) between the participants who agreed (agree/somewhat agree) with the use of text messaging to communicate with a professional to receive help to manage anxiety and those who disagreed (disagree/somewhat disagree). Similarly, there were no statistically significant differences between group mean scores for psychological openness (*P*=.78), help-seeking propensity (*P*=.93), indifference to depression stigma (*P*=.67), and IASMHS scores (*P*=.94) between the participants who agreed (agree/somewhat agree) with the use of text messaging to communicate with a professional to receive help to manage depression and those who disagreed (disagree/somewhat disagree). In addition, the results of the sensitivity analysis revealed no statistically significant difference between group mean scores, in the aforementioned categories, between the participants who agreed (agree/somewhat agree) and those who did not indicate agreement (disagree/somewhat disagree/undecided) with the use of text messaging to communicate with a professional to receive help to manage anxiety (psychological openness: *P*=.88; help-seeking propensity: *P*=.93; indifference to anxiety stigma: *P*=.32; and IASMHS scores: *P*=.55) and depression (psychological openness: *P*=.62; help-seeking propensity: *P*=.56; indifference to depression stigma: *P*=.81; and IASMHS scores: *P*=.59).

**Table 2:** Inventory of Attitudes Toward Seeking Mental Health Services factor scores by agreement with using text messaging to communicate with a professional to receive help for managing anxiety.

Factor	Agree (n=49), mean score (SD)	Disagree (n=41), mean score (SD)	Mean difference (95% CI)
Psychological openess	23.9 (5.0)	23.9 (4.0)	0.0 (−1.9 to 1.8)
Help-seeking propensity	26.1 (5.1)	26.5 (4.8)	−0.4 (−2.5 to 1.6)
Indifference to anxiety stigma	23.7 (6.2)	25.1 (5.9)	−1.4 (−3.9 to 1.1)
Inventory of Attitudes Toward Seeking Mental Health Service total	73.7 (12.9)	75.5 (11.3)	−1.9 (−7.0 to 3.2)

**Table 3:** Inventory of Attitudes Toward Seeking Mental Health Services factor scores by agreement with using text messaging to communicate with a professional to receive help for managing depression.

Factor	Agree (n=43), mean score (SD)	Disagree (n=43), mean score (SD)	Mean difference (95% CI)
Psychological openess	24.2 (5.1)	23.9 (4.1)	0.3 (−1.7 to 2.3)
Help-seeking propensity	26.4 (4.7)	26.3 (5.0)	0.1 (−2.0 to 2.2)
Indifference to depression stigma	23.8 (6.5)	24.3 (6.1)	−0.6 (−3.3 to 2.1)
Inventory of Attitudes Toward Seeking Mental Health Service total	74.4 (12.8)	74.6 (12.0)	−0.2 (−5.5 to 5.1)

### Concerns About Text Messaging

More than half of the respondents (56/101, 55.4%) indicated having concerns about using text messaging to communicate with a professional. The most common themes identified from responses to the question, “Do you have any concerns about using text messaging to communicate with a professional?” are presented in Table 4. A total of 78 responses were coded into themes. Most of the concerns (73/78, 94%) centered around the following themes: privacy and confidentiality (33/78, 42%), the impersonal feel of communicating by text messaging (17/78, 22%), possible miscommunication (16/78, 21%), and belief that the mode is insufficient for treatment (7/78, 9%).

**Table 4:** The most common concerns about using text messaging to communicate with a professional (N=78).

Themes^a^	Value, n (%)	Examples
Privacy and confidentiality	33 (42)	* “There are risks with sending sensitive information in text messages such as being mistakenly sent to the wrong person, someone other than the professional seeing/reading my messages, and someone other than the professional sending them in response to my messages.”* [Participant, 34 years old] *“Security of the content of the text messages.”* [Participant, 31 years old] *“Lack of privacy. The government has been known to search the cell phones of law abiding individuals for ridiculous reasons.”* [Participant, 58 years old]
Impersonal feel	17 (22)	*“Lack of intimacy with counselor. How can healing take place without a relationship?”* [Participant, 29 years old] *“Too impersonal—and tone is too difficult to determine and you cannot read compassion.”* [Participant, 48 years old] *“Not personal enough.”* [Participant, 62 years old]
Miscommunication	16 (21)	*“I believe body language is really important in communication. Text messaging doesn’t allow for the counselor to observe body language. Writing can also sometimes be misunderstood by the reader.”* [Participant, 29 years old] *“It’s hard to convey emotions via text message.”* [Participant, 34 years old] *“Words are just 35% of communication.”* [Participant, 65 years old]
Insufficient mode for treatment	7 (9)	* “Their response time...with video conferencing you can get immediate feedback versus waiting for someone to respond [via text] which may increase my anxiety.”* [Participant, 34 years old] *“It depends on the severity of the issue I am working through. I believe there are instances were text messaging is inappropriate or insufficient.”* [Participant, 32 years old] *“I’m not sure if [text messaging] would be as effective.”* [Participant, 33 years old]

^a^A total of 78 responses were coded into themes, however only the most common themes are presented in the table.

## Discussion

### Principal Findings

To our knowledge, this preliminary study was one of the first to measure the acceptability of using text messaging to deliver mental health care to African American women. The results of this study showed that less than half of respondents endorsed the use of text messaging to communicate with a professional to receive help to manage anxiety and depression. No statistically significant associations were found between age and agreement with the use of text messaging. Approximately half of the women agreed that having the option to use text messaging to communicate with a professional if they are dealing with anxiety or depression would be helpful. However, more than half of respondents indicated having concerns about using text messaging to communicate with a professional. No statistically significant association was found between age and having concerns about using text messaging to communicate with a professional.

The results revealed that African American women have favorable views toward seeking mental health services, comparable with non–African American women [27]. Our findings are contrary to the results of a previous study by Watson and Hunter who found that African American women have less favorable attitudes toward professional help seeking than their non–African American counterparts [3]. However, the differences in reported results between the studies may be because of significant differences in age and education level between the study samples. The mean age of the women in the study by Watson and Hunter [3] was 20.9 years, and the majority of participants (92.6%) reported attending a 4-year university. In comparison, the mean age of the women in our study was 38.9 years, and the majority of participants (85.2%) had at least a bachelor’s degree. Therefore, the 18-year difference in mean age between the study samples and the difference in education level could contribute to the contrasting findings.

An exploration into the reason for low acceptance of text messaging was conducted by analyzing group IASMHS factor scores by the level of agreement with using text messaging to communicate with a professional to receive help for managing anxiety and depression. Findings showed that there were no statistically significant differences between group mean scores for any of the factors. One might expect to see a significant difference in psychological openness, help-seeking propensity, indifference to anxiety stigma, or indifference to depression stigma between the participants who agreed (agree/somewhat agree) with the use of text messaging and those who disagreed (disagree/somewhat disagree). Specifically, the authors expected that those who indicated acceptance of the use of text messaging would have higher scores for all factors than those who did not. These findings could be interpreted as indicating that the reason for low acceptance is not because of a difference in attitudes toward seeking mental health care but because of the modality used to do so.

The most common concerns respondents had were about privacy and confidentiality, the impersonal feel of communicating by text messaging, possible miscommunication, and belief that the mode is insufficient for treatment. For example, regarding privacy and confidentiality, 1 respondent stated:

There are risks with sending sensitive information in text messages such as being mistakenly sent to the wrong person, someone other than the professional seeing/reading my messages, and someone other than the professional sending them in response to my messages.

Concerns around privacy and confidentiality must be addressed for the successful implementation of mHealth interventions for African American women [23]. Future studies should provide clear communication to participants about who they will receive text messages from, who will have access to the text messages, and information on how the data will be protected. The Health Insurance Portability and Accountability Act of 1996 (HIPAA) sets national standards to protect sensitive patient health information. Researchers and clinicians should ensure that transmission and storage of text messages that contain electronic protected health information are HIPAA compliant.

Furthermore, concerns around the impersonal feel and possible miscommunication must be considered. One respondent noted:

[text messaging is] too impersonal—and tone is too difficult to determine and you cannot read compassion.

Another respondent voiced concerns about possibly being misunderstood by stating:

I believe body language is really important in communication. Text messaging doesn’t allow for the counselor to observe body language. Writing can also sometimes be misunderstood by the reader.

It is important that participants feel connected to the person they are disclosing sensitive information to, especially within a population where mental illness is highly stigmatized. Feelings of disconnection and being misunderstood are counterproductive to treatment. Although text messaging is known to increase the feeling of connectedness between patient and mental health professionals, successful use is limited to simple messages (eg, supportive messages to prevent suicide attempt) and not real-time prolonged conversation to manage a current episode (eg, panic attack) [25].

Finally, text messaging may not be appropriate to use in all situations. One respondent stated:

It depends on the severity of the issue I am working through. I believe there are instances were text messaging is inappropriate or insufficient.

Furthermore, a unique finding was that the use of text messaging may actually increase anxiety because of a lag in response time. A participant noted the following concern:

Their response time...with video conferencing you can get immediate feedback versus waiting for someone to respond [via text] which may increase my anxiety.

The combination of concerns around privacy and confidentiality, in addition to the impersonal feel, fear of miscommunication, and view of text messaging as an insufficient mode for treatment, presents a significant challenge to the use of this modality for effective treatment of anxiety or depression. Although text messaging is convenient, it may not be easily adopted or sustainable to use to converse with clients regarding their anxiety or depression. A systematic review on the use of text messaging for mental health care concluded that “due to the simplicity of its content, text messaging cannot be used as a remote counseling tool;” however, previous studies have successfully used text messaging as an adjunct to in-person treatment [25]. Text messaging may be considered for symptom monitoring and appointment, medication, and *homework* reminders (eg, CBT activities), which may help to reduce no-show rates, improve medication adherence, and increase the likelihood of completing *homework* assigned by the mental health professional [34-36]. A study by Aguilera et al found that daily automatic text message–based mood ratings can be used as a proxy for the Patient Health Questionnaire-9 depression screener [37]. This may be beneficial for tracking depression severity to identify trends and adjust treatment plans as needed.

### Limitations

The main limitations of this exploratory study were recruitment method and sample size. Participants were recruited through convenience sampling and encouraged to share the survey email or social media posts with their networks. Although no PII was collected in the survey and respondents accessed the survey through an anonymous link, social desirability and other selection biases could have resulted if the respondent personally knew the first author.

Furthermore, the sample size of 101 respondents is small for this cross-sectional survey and consisted of mostly younger (<50 years) and highly educated women (more than 85% had at least a bachelor’s degree). This limits the generalizability of the findings. Although stigma may continue to be a barrier for highly educated African American women, access to mental health services, insurance coverage, and health literacy may be less of an issue for this group.

### Conclusions and Future Directions

Owing to the high smartphone ownership by African American women (80%) [24], there is a great opportunity to use mobile technology to provide mental health care. A *one-size-fits-all* approach to designing telehealth interventions to help African American women manage anxiety or depression may lead to more options but continued disparity in receiving mental health care. This study adds to the literature by providing insight into the attitudes of African American women toward seeking mental health services to manage anxiety and depression and the acceptability of using text messaging to communicate with a professional to receive help for managing anxiety and depression. Although the use of text messaging was not highly endorsed by African American women as an acceptable mode to converse with a professional (<50% endorsed), our prior work found that mobile video calls were viewed favorably by the majority of respondents (>70% endorsed) [22]. Concerns around privacy, confidentiality, and the impersonal feel of communicating about sensitive issues via text messages must be addressed for successful participation in text message–based interventions among this population. However, it may be used as an adjunct to other methods for remote counseling (eg, video call and voice calls) [38].

The findings of this study demonstrated the need for additional research into the use of mobile technology to provide African American women with more accessible and convenient options for mental health care. More research is needed to determine whether having a preexisting relationship with a professional (eg, face-to-face sessions in the past) impacts acceptance and use of the technology to receive professional support. Future work will include relaunching the survey to a larger and more generalizable sample. Questions will be added to screen for the presence and severity of depression and anxiety and to collect data on previous mental health services utilization and history of mental illness.

## Abbreviations

CBT: cognitive behavioral therapy
HIPAA: Health Insurance Portability and Accountability Act of 1996
IASMHS: Inventory of Attitudes Toward Seeking Mental Health Services
mHealth: mobile health
PII: personally identifiable information
